# Integrating Complementary and Alternative Medicine with Primary Health Care

**DOI:** 10.1155/2013/948308

**Published:** 2013-07-14

**Authors:** Annie Shirwaikar, Raghavan Govindarajan, Ajay Kumar Singh Rawat

**Affiliations:** ^1^Gulf Medical University, Ajman, UAE; ^2^Dabur International Ltd., Jebel Ali Free Zone, Dubai, UAE; ^3^Pharmacognosy Department, National Botanical Research Institute, Lucknow, India

Complementary/alternative medicine (CAM) has been described as “diagnosis, treatment, and/or prevention which complements mainstream medicine by contributing to a common whole, satisfying a demand not met by orthodoxy, or diversifying the conceptual frameworks of medicine” [1]. Approximately, 1500 articles on CAM are published annually in the literature covered by MEDLINE [2]. In the United Kingdom, the market for herbal and homoeopathic remedies and aromatherapy oils increased by 41% between 1992 and 1996 [3]. In Germany, a herbal remedy (St. John's wort) is now the most frequently prescribed drug for depression. In the USA, the sales of St. John's wort rose by 2800% between 1997 and 1998, and the total market for medicinal botanicals was worth US$ 3.87 billion in 1998 [4]. Most experts agree that the interest in and practice of CAM are driven mainly by consumer pressure. A 1990 national survey of alternative medicine prevalence in US, costs, and patterns of use [5] demonstrated that alternative medicine has a substantial presence in the US health care system. Data from a survey in 1994 [6] and a public opinion poll in 1997 [7] confirmed the extensive use of alternative medical therapies in the United States. An increasing number of US insurers and managed care organizations now offer alternative medicine programs and benefits [8]. The majority of US medical schools now offer courses on alternative medicine [9]. In western Europe and Australia, 20–70% of the population regularly use complementary and alternative medicine [10, 11]. In the USA, it was estimated in 1992 that at least one in three Americans utilized one of those methods, and the number of annual visits to providers of alternative medicine exceeds the number of visits to all primary care physicians [12]. These therapies include acupuncture, chiropractic, herbal medicine and dietary supplements, nutraceuticals, homeopathy, mind-body techniques, spirituality, and faith healing, massage, and therapeutic touch. In a 1998 follow-up study, the percentage of CAM patients had increased to 42% of the US population [13]. Subsequent analyses showed that 67.6% of respondents had used at least one CAM therapy in their lifetime. This trend suggests a continuing demand for CAM therapies that will affect health care delivery for the foreseeable future [14]. The WHO report of 2002 states that at least 70% of the world population still believes in alternative medicine, and therapies which include homeopathy, Ayurveda, Siddha, Unani, Amchi, acupuncture, aromatherapy, herbal medicine in general, dietary supplements, nutraceuticals, Yoga, mind-body techniques, spirituality and faith healing, and massage. The current trend is indicative of a continuing demand for CAM therapies which will definitely have its impact on health care delivery in the future [15].

The term primary healthcare (PHC) has been interpreted in different ways. At its core, PHC is defined as a set of universally accessible services that promote health, prevent disease, and provide diagnostic, curative, rehabilitative, supportive, and palliative services. At the heart of PHC, reform is the goal to establish a holistic health and social-service system that emphasizes health promotion and disease prevention. With an emphasis which is holistic and more personalized, unlike the symptomatic approach of the orthodox system of medicine, CAM should be able to complement the goals of PHC per se. Many of the concepts inherent to CAM are consistent with those recommended by already established PHC services. Integrating the health services may serve to enhance health care equity wherein all individuals may have access to a full range and combination of health care services that can contribute to reduced sickness and an increased health-related quality of life. To support and facilitate this endeavour, there is a need, as well as potential for an improved public health mandate, to monitor and promote the integration of CAM with PHC.

Despite a few attempts, it has yet to be established how to integrate CAM therapies into the conventional medical system in a systematic way. A logical first step in this direction of integration is to establish guidelines for the proper integration of CAM into primary care, supported by appropriate research and clinical experience. Unfortunately, the research data on this issue are quite limited. Recently, in the USA, the Federation of State Medical Boards developed and adopted new model guidelines for the use of complementary and alternative therapies in medical practice [16].

 A summary of 26 surveys across 13 countries concluded that the prevalence of CAM use by cancer patients overall was 31.4% (range: 7% to 64%) [17]. Most cancer patients combine CAM with conventional therapy [18, 19]. Oncologists are becoming increasingly aware that patients use CAM, yet few oncologists discuss these therapies with patients. Instead, the established medical community is demanding regulation and evaluation of CAM [20]. Some groups insist that CAM poses serious health risks and cite poor outcomes for patients who reject proven conventional cancer treatment for CAM approaches [19]. The increasing interest in CAM among cancer patients may be due to limitations of conventional cancer treatment, increased advertising and media coverage of CAM, or the desire for holistic or natural treatments. Cancer patients want more information, and some patients believe that access to CAM should be part of standard cancer treatment [21]. As cancer incidence increases and survival time lengthens, the population seeking information about an access to CAM is likely to increase. A review examining the overlap between participation in allopathic breast cancer early detection activities and CAM use has been presented.

Herbal medicine (HM) is one of the most widely used complementary and alternative medicine (CAM) therapies used throughout the world. In many countries, HM has a long tradition and the knowledge about local medical plants is ingrained into cultural memory. The WHO estimates that 70–90% of the rural population in developing countries use HM to meet, in part or completely, their health needs [22]. Also, in many developed countries, HM as an element of CAM is highly popular. Therefore, HM is recognized as an essential component of primary healthcare by the WHO [22]. In this context, the perspectives and experiences of patients using HM in a primary care context have also been presented. Herbal medicines automatically take us to traditional Chinese medicine (TCM) which has always been popular in China and also among the Chinese in other parts of the western world. Hence, a study describing the utilization of TCM services by those attending the community health centres has been presented in detail including the prevalence and frequency of TCM use and health-related characteristics of TCM users. Unique dual medical system in the Republic of Korea has resulted in the emergence of dual licensed medical doctors (DLMD) who have both traditional Korean medicine (KM) and western medicine (WM) licenses. There have been few studies on DLMD in spite of their growing number and importance within the medical system. Hence a survey of the current status and attitudes of DLMD to assess their role in integrative medicine has been presented. 

There are two major ways of bioprospecting natural products for investigation. First, the classical methods that rely on phytochemical factors, serendipity, and random screening approaches. Second, the use of traditional knowledge and practices as a drug discovery engine, which is known as the ethnopharmacological approach, which is a time and cost effective approach and could lead to better success than random screening [23, 24]. Traditional methods, for example, Chinese medicine, Japanese Kampo, and Indian Ayurveda, are becoming important bioprospecting tools [25]. Ethnopharmacological validations of some herbal/traditional medicine using modern pharmacological tools have been presented.

Most of the herbal drugs produced currently in the developing countries lack proper quality specification and standards and therefore have no consistency in quality in batch to batch products. Most of these drugs do not have well-defined and characterized composition. A well-experienced traditional physician in the past used to have specific knowledge and special ability to collect the right plants having the therapeutically useful agents from certain specific habitats. These experienced medicinal plant collectors had intimate knowledge of plant species and could identify therapeutically effective plant from a population of a species. With such unique expertise, they were able to maintain certain level of standards in the therapeutic quality of the herbal drugs. There had been a decline or almost extinction of such experienced plant collectors by the turn of the 20th century itself due to a variety of reasons. One of the reasons was the transformation of traditional medicine from the individualized system to a commercial manufacturing system. It is now well known that the therapeutic activity of a medicinal plant is due to the presence of certain biologically active chemical constituents, which are either primary or secondary metabolites. The expression of many of these compounds particularly those of the secondary metabolite category is controlled and conditioned by a variety of factors such as its genetic predisposition, habitat of the plant, agroclimatic conditions, season, and also the stage of growth and development of the plant. Finally, a method for quality evaluation of herbal drugs has also been presented. 

Thus, the integration of the traditional and complementary medicine into PHC involves, understanding the CAM, knowing the need gaps in the existing system of medicine, validation of the activity of the traditional medicine to induce confidence in the practitioner/patient, and finally the chemical standardization of the drugs to ensure proper quality of the herbal drugs.

*Annie Shirwaikar* *Annie Shirwaikar* *Raghavan Govindarajan* *Raghavan Govindarajan* *Ajay Kumar Singh Rawat* *Ajay Kumar Singh Rawat*
